# Clinical translation and implementation of optical imaging agents for precision image-guided cancer surgery

**DOI:** 10.1007/s00259-020-04970-0

**Published:** 2020-08-12

**Authors:** F. B. Achterberg, M. M. Deken, R. P. J. Meijer, J. S. D. Mieog, J. Burggraaf, C. J. H. van de Velde, R. J. Swijnenburg, A. L. Vahrmeijer

**Affiliations:** 1 Department of Surgery, Leiden University Medical Center, Leiden, The Netherlands; 2 Centre for Human Drug Research (CHDR), Leiden, The Netherlands; 3 Department of Surgery, Cancer Center Amsterdam, Amsterdam UMC, University of Amsterdam, Amsterdam, The Netherlands

**Keywords:** Clinical translation, Fluorescence-guided surgery, Image-guided surgery, Precision surgery

## Abstract

**Introduction:**

The field of tumor-specific fluorescence-guided surgery has seen a significant increase in the development of novel tumor-targeted imaging agents. Studying patient benefit using intraoperative fluorescence-guided imaging for cancer surgery is the final step needed for implementation in standard treatment protocols. Translation into phase III clinical trials can be challenging and time consuming. Recent studies have helped to identify certain waypoints in this transition phase between studying imaging agent efficacy (phase I–II) and proving patient benefit (phase III).

**Trial initiation:**

Performing these trials outside centers of expertise, thus involving motivated clinicians, training them, and providing feedback on data quality, increases the translatability of imaging agents and the surgical technique. Furthermore, timely formation of a trial team which oversees the translational process is vital. They are responsible for establishing an imaging framework (camera system, imaging protocol, surgical workflow) and clinical framework (disease stage, procedure type, clinical research question) in which the trial is executed. Providing participating clinicians with well-defined protocols with the aim to answer clinically relevant research questions within the context of care is the pinnacle in gathering reliable trial data.

**Outlook:**

If all these aspects are taken into consideration, tumor-specific fluorescence-guided surgery is expected be of significant value when integrated into the diagnostic work-up, surgical procedure, and follow-up of cancer patients. It is only by involving and collaborating with all stakeholders involved in this process that successful clinical translation can occur.

**Aim:**

Here, we discuss the challenges faced during this important translational phase and present potential solutions to enable final clinical translation and implementation of imaging agents for image-guided cancer surgery.

## Introduction

Tumor-targeted imaging has changed our view on cancer diagnostics and therapy in the past two decades [1, 2]. These modalities provide feedback on the tumor’s biomolecular features combined with high spatial and, specifically for fluorescence-guided surgery (FGS), high temporal resolution. Our group reported on the challenges and limitations of optical image-guided cancer surgery in 2013 [3]. Now, several years later, we aim to consider the translational challenges that still exist and those which have presented themselves over the past years.

The potential for cancer-detection methods and vital-structure discrimination using exogenous contrast agents, or imaging agents, have been described extensively over the past years [4–6]. It was only until 2000 when PET/CT was granted the “invention of the year” award by the *Time Magazine* [7] that spatial and molecular information could be integrated into one platform. Shortly thereafter, fluorodeoxyglucose positron emission tomography ([^18^F]-FDG-PET) gained FDA approval in 2002 [8] and paved the way for these new molecular imaging approaches in many cancer types [9–11]. Further insight in protein engineering and molecular cell biology launched a search for suitable biomarkers for tumor-specific molecular imaging [12, 13]. The knowledge acquired by antibody and ligand-mediated PET imaging [2] was early on adopted by adding a fluorophore to these binders enabling tumor-specific fluorescence-guided surgery (TS-FGS) in the near-infrared spectrum (~ 700–800 nm) [14, 15]. This field specifically has seen great advancements from 2013 onwards as antibody-mediated TS-FGS paved the way for the clinical translation of tumor-specific imaging agents in recent years [16, 17]. In parallel, the dissemination of knowledge and the clinical availability of fluorescence imaging systems [18] resulted in a surge of clinical studies with non-tumor-specific fluorescent dyes [19–21]. Technical variables related to optical or fluorescence imaging itself have been described extensively by others (e.g., camera standardization, signal quantification, efficacy of the imaging agent, and trial design) [6, 16, 22]. However, phase III clinical trials are needed to study patient outcome and enable widespread acceptance and implementation of TS-FGS.

When a multicenter phase III trial is initiated, meticulous preparations of the trial promote standardized data acquisition (e.g., reproducible data) to further facilitate general acceptance and implementation of FGS. Only a handful of randomized phase III trials with tumor-specific fluorescence imaging agents have been initiated in recent years and many more with non-tumor-specific fluorescent dyes like indocyanine green (ICG) [19, 23, 24]. These studies have helped to identify a unique set of variables specifically related to transferring knowledge of the translation and implementation of FGS into the clinic. Identifying these factors of variability before implementing TS-FGS into centers that are not primarily research-focused, though pivotal to creating multicenter data, is important for data quality. Here, we aim to discuss several waypoints between phase II studies and final translation of TS-FGS, which help to increase standardized data acquisition. More specifically, we emphasize the importance of training clinicians, setting up a multidisciplinary study approach and we propose the term clinical significant event to be used as a study endpoint in future phase II–III trials. Finally, we will contemplate on future opportunities in clinical implementation of FGS for tumor-targeted cancer surgery.

## Defining the clinical and imaging framework

### Clinical significant event

**Fig. 1:**
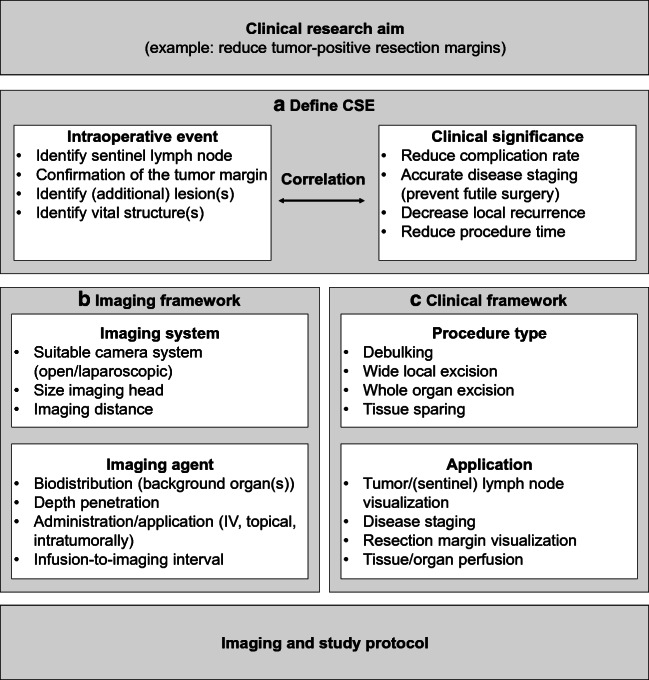
Flowchart for defining a CSE. When a phase III trial is initiated, the clinical research aim is first formulated. In this example, the aim is to reduce tumor-positive resection margins in a given tumor type. To answer the research question, a CSE is defined based on the correlation between an intraoperative imaging event and its clinical significance. This correlation should be well studied and its significance preferably discussed with regulatory authorities and specialists in the field. Defining a CSE automatically leads to both an imaging and clinical framework. The imaging framework (b) includes variables of the imaging agent and imaging system. The clinical framework (c) describes the procedure type and clinical application. Predefining these variables for each study can promote capturing a CSE with NIRF imaging, thus help answering the research question

Assuming efficacy and safety of an imaging agent is determined earlier, a phase III study is initiated when a valid clinical research question is established (Fig. 1a). In order to show patient benefit, the impact of using FGS needs to be directly correlated to patient outcome [25]. For example, demonstrating a relation between tumor-free resection margins and improved patient survival. In the past, these surrogate endpoints have been accepted by regulating authorities as study endpoints [25]. However, current day oncology care is becoming increasingly complex, with surgery being only a part of the entire treatment plan. Establishing a specific surgical event as the game-changing event in the survival is rather challenging, though needed for regulatory approval. Identifying and accurately describing these events, more like a surgical-biomarker, might help us to better understand the impact of certain decisions within a surgical procedure. However, identifying these biomarkers or even proxy biomarkers is non-trivial. Secondly, implementing them as trial endpoints would require consent of regulators. In this perspective, a valuable example can be found in the clinical trial studying the impact of the tumor-specific imaging agent 5-aminolevulini acid (5-ALA) in patients with malignant gliomas [26]. Here, surgical radicality was successfully used to measure disease-free and overall survival, resulting in FDA approval of the drug in 2017, some 11 years after the study was published.

Although there is a need for a general applicable clinical trial endpoint for TS-FGS trials, the variation between surgical procedures, tumor types, and surgical site creates a difficulty in defining such an endpoint [22]. We advocate to define a clinical significant event (CSE) as trial endpoint for FGS trials (Fig. 1a). CSE are defined as the occurrence of a predefined surgical event provided by imaging data. The surgical event (e.g., tumor-margin detection) has an established correlation, or clinical significance, with the outcome (e.g., increased disease-free survival). Additional optimal clinical trial endpoints could be explored for each tumor type, with or without neoadjuvant therapy, and specific to the procedure. Using relatively short-term endpoints as decreased incidence of re-excision, reoperation, and/or iatrogenic injury, improved morbidity and reduced local recurrence rates could demonstrate clinical benefit of TS-FGS to patients. Defining a CSE automatically leads to an imaging framework (Fig. 1b), along with the imaging data from the phase II study. The clinical framework (Fig. 1c) provides guidelines on which patients to include (e.g., tumor type, disease stage, procedure). Although phase II trials are commonly not powered to prove that an intervention results in a better clinical outcome, ideally, the CSE is studied in these phase II trials to get an understanding of its feasibility and variability. This would enable designing properly powered pivotal trials.

### Imaging systems for clinical translation

For optimal imaging data in a phase III clinical trial, the imaging agent should be matched with a NIR fluorescent imaging system based on optical properties (choice of excitation source and filters to optimize fluorescent yield) and technical properties (procedure type). The trial team should take into account that all fluorescence imaging systems differ in their operational characteristics [18], signal acquisition, and post-processing of images. One should be forethoughtful that different camera systems, optimized for the same wavelength, might produce different images from the same imaging agent used in similar concentrations and solvents [27]. Ex vivo or in vitro comparison of clinical imaging systems with a tumor-specific imaging agent, especially laparoscopic systems, rarely leads to adequate and comparable data. Moreover, due to different legislations, the speed of development of imaging systems significantly exceeds that of imaging agents [28]. As a consequence, soft- and hardware might change during the transition from preclinical to phase II–III studies and can significantly influence the image acquisition [29].

To date, a selection of NIR imaging systems are commercially available for intraoperative use and are designed for either open, laparoscopic, or robotic procedures and in some cases endoscopic examinations [30, 31]. The majority of which are calibrated to deliver optimal image quality around the excitation and emission spectrum of ICG (Ex_max_ ~ 780 nm, Em_max_ ~ 810 nm), while other NIR imaging systems have their sensitivity for certain fluorophores [16, 32] and some systems allow multi-fluorophore imaging.

Although most of the conjugatable fluorophores used to enable TS-FGS are excited around the same wavelength as ICG, the fluorescent yield is often suboptimal. Tumor-specific imaging agents are usually present in the tissue of interest at nanomolar concentrations, which requires sensitive cameras with low detection thresholds. Minimally invasive surgery eliminates some of the disadvantageous imaging conditions (e.g., less surrounding light, closer working distance). Yet, current laparoscopic NIR fluorescence imaging systems have a significantly lower fluorescent yield as compared with open systems, mainly due to safety limitations concerning the power of the excitation laser. Bridging this technical gap is one that can only be achieved by collaborating intensively with optical engineers, camera developers, preclinical scientists, and surgeons. Only if the imaging agent, more specifically the fluorophore, is well matched with the clinical imaging system can standardized reporting really tell us something about the efficacy of the imaging agent itself (phase II study) and its potential for clinical use (phase III study). Standardized back-table pathology plays an important role in studying the biodistribution of an imaging agent in the tissue of interest [33], but lacks the majority of information about the potential clinical, intraoperative use. A suboptimal image is not necessarily caused by dysfunction of the imaging agent rather than a suboptimal combination of one of the elements in this “imaging cascade” (binding moiety, fluorophore, tissue type, dosing, imaging interval, camera system, camera use, post-processing, image interpretation).

### Quality: train the clinician

Performing TS-FGS requires background knowledge about the physiological behavior of NIR light, the pharmacodynamics, and pharmacokinetics of the imaging agent, the camera setup, and the intraoperative workflow. For TS-FGS to be applied in a standard clinical fashion, data acquisition and interpretation are both executed by a clinician. All these assets are therefore of influence on data quality and reproducibility. Although most clinically available camera systems are almost “plug-and-play” platforms, running a phase III trial, thus hands-on performing NIR fluorescence imaging, requires a surgical workflow designed specific for the trial. One should be aware that the combination of clinician and camera system together makes the imaging platform. Even before the first patient is included, participating surgeons need to be thoroughly trained. Especially in later phase trials where the influence of FGS on clinical decision-making or patient outcome is studied, data interpretation by the end-user is vital for the study’s overall quality, for example, dry-lab session to get familiar with the imaging system and acquire hands-on experience on the effect of imaging distance, camera angle, and overlaying tissue. Tissue perfusion studies with non-targeted dyes (e.g., anastomosis perfusion) or sentinel lymph node localization supervised by an expert can be used for this training purpose since the clinical application is relatively well studied in retrospective cohorts [34, 35]. This way, surgeons can acquire hands-on knowledge with FGS and, if possible, be proctored by an experienced clinician.

## Teamwork

**Fig. 2:**
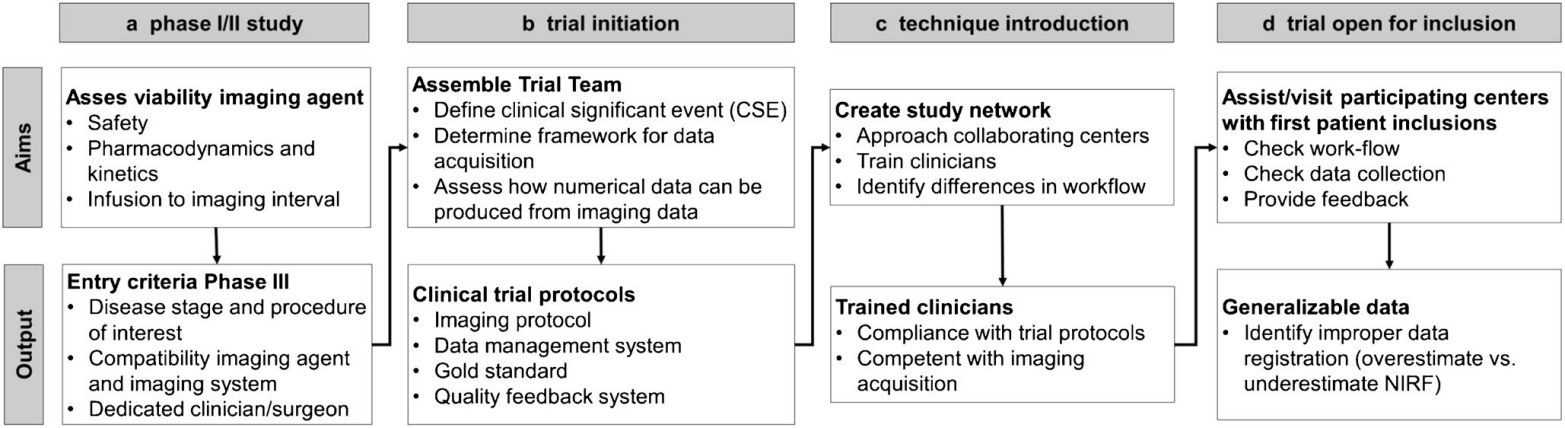
Waypoints for clinical introduction and final translation of fluorescence-guided surgery

For TS-FGS to impact standard patient care, study data should be comparable and generalizable amongst clinicians, thus requiring minimal interobserver variability. Currently, most translational studies are performed in university medical centers or training hospitals, whereas 80–90% of cancer patients receive their care in a community hospital [36]. Involving motivated clinicians outside the centers of expertise, and therefore creating a network, promotes the translatability of TS-FGS. When a multicenter phase III trial is initiated, one should take in mind that using FGS is a completely new entity to most clinicians that participate in the study. Assembling a dedicated trial team (overseen by a motivated coordinator) can help to move from phase II, where the imaging agent takes center stage, to phase III where patient benefit is studied. Certain waypoints can be identified between these two studies phases (Fig. 2).

**Fig. 3:**
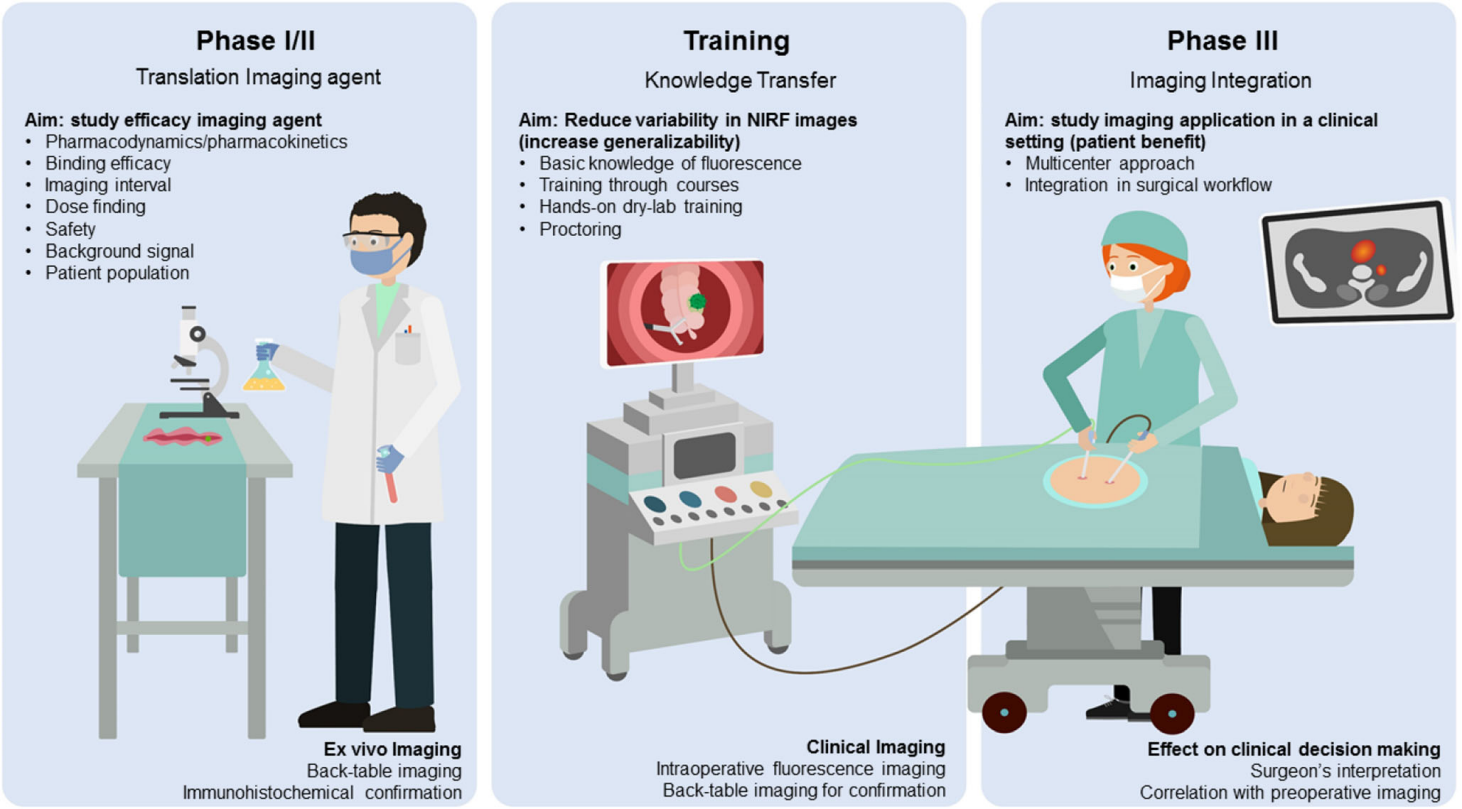
Roadmap to precision surgery. Early clinical translation (phase I/II) of imaging agents for tumor-specific fluorescence-guided surgery has well-established study endpoints, focused on the efficacy and safety of the substance. However, proving patient benefit in phase III studies introduces the clinician and its ability to interpret and act upon imaging information as a variable. Therefore, training clinicians from all participating centers in performing FGS is vital to enable adequate data acquisition and increase compliance with the imaging protocol. For FGS to impact patient care, clinicians should be able to adequately interpret images and integrate fluorescence imaging into their surgical workflow. Furthermore, integration of tumor-specific preoperative (PET) imaging can create a case-by-case dashboard of both biomolecular and spatial information to enable precision surgery

Based on the CSE, imaging agent, and NIR fluorescence camera system, the trial team designs the imaging protocol. Technical feasibility and data quality prerequisites need to be discussed between the surgeon and data specialists. Both should be aware on how imaging data is acquired in the operating room, but also how it is eventually transferred into numerical data and further analyzed to answer the research question. This framework is then used by the surgeon to finalize the imaging protocol, taking in mind the technical specifications of the imaging system (e.g., working distance, field of view) and the procedure of interest (e.g., location of interest, background organs, imaging time-points) [32]. In parallel, the data scientist and pathologist establish the reference standard for the trial. A central pathology review in multicenter setting is preferential, also to promote generalizability of the data. When fully established, the protocols can be used to train clinicians in collaborating centers (Fig. 3). Involving participating clinicians early on and shadowing them while performing the procedure of interest helps to further identify potential center-specific bottlenecks.

After the first inclusions, quality control and quality feedback by an expert of the field and study coordinator are important for correct interpretation of study results. Low compliance with the imaging protocol and data processing can encourage over and/or underestimation of the study results [37]. Periodic evaluation of study data by the trial team and the participating center can promote timely identification of faulty data. When performing a large multicenter surgical trial, quality feedback and training can have an educational effect on a national level [38].

## Where does FGS fit into the imaging spectrum?

Though FGS provides new perspectives for surgeons, combining fluorescence imaging with other tumor-specific imaging modalities might further increase accurate patient selection and help to identify the population who might truly benefit from TS-FGS. FGS has many advantages over conventional preoperative imaging (no radiation, real-time, higher spatial resolution). Nonetheless, the majority of multidisciplinary team meetings still predominantly rely on 2D, anatomical, gray-scale images for disease staging and defining the treatment plan, in some cases combined with circulating biomarkers (e.g., CA19-9, CA125, PSA, CEA) [39, 40]. Daily care treatment standards are established using large data sets often based on non-specific preoperative imaging and regular visual intraoperative inspection. The effect of restaging patients during surgery using a new imaging modality as TS-FGS and changing treatment policy accordingly is unknown and therefore needs to be studied. In this perspective, the surgical field might benefit from the growing knowledge in tumor-specific PET agents being studied. In some cases, the same targeting peptide is subsequently used for preoperative PET imaging and could provide biomolecular feedback of the tumor and potential distant metastases prior to surgery. In both fields, interesting questions remain regarding study design; if a patient undergoes both the reference standard imaging modality and tumor-targeted imaging modality, should one receive a standard-of-care treatment afterwards regardless of the information provided by the new imaging modality? Or should the treatment be adjusted according to additional information from an experimental imaging modality, where the effect of the adjustment is largely unknown?

Multiplex imaging with an array of biomarkers is not a new idea, but is often done using the same imaging approach. Multimodal molecular imaging using different biomarkers will provide both molecular and spatial information. Such a multimodal imaging approach can provide the spatial information needed to plan or execute surgical treatment where in vitro diagnostics often stay behind. Also, blood drawn from the circulation is inherent to sample bias, created by a pooled sample of all biomarkers present in the circulation and is therefore lacking the spatial information.

In this perspective, staging laparoscopies are routinely performed, and FGS is expected to be of added value there. However, multimodal molecular imaging should be able to provide this same information non-invasively, potentially preventing invasive procedures. This emphasizes the need for accurate preoperative staging with tumor-targeted imaging modalities with radionucleotides or for instance ultrasound to acquire this information beforehand. This enables the clinician to use TS-FGS to its best and, when needed, can be used for localization of the target lesion, check for distant (e.g., peritoneal) disease, or confirm a complete resection (Fig. 3). Preferably, changing of treatment plans should be minimal during surgery. Examples can be found from ICG-technetium imaging for sentinel lymph node biopsies in head and neck tumors [41], where ICG is solely used to localize the target node, visualized with SPECT imaging a day or hours prior to surgery. Hopefully, the integration of different imaging modalities will bring about a paradigm shift from a predominantly anatomy-based staging approach, towards a biology-based approach in the field of cancer surgery [42, 43].

## Outlook

Other opportunities and challenges of FGS lay in the increased use of minimally invasive surgery and neoadjuvant treatment. Surgeons lack the tactile information regarding benign or malignant tissue with robotic-assisted and laparoscopic surgery. Feedback from a fluorescent imaging signal could provide tactile information visually. Moreover, after neoadjuvant treatment, tumor-specific imaging enables a more accurate assessment of the treatment response and active regions of the tumor. With neoadjuvant treatment being applied in a growing number of cases, the need for tumor-specific molecular imaging becomes therefore increasingly evident. In this perspective, the better spatial resolution of FGS over PET may allow for more accurate assessment of vital tumor tissue. One should be cautious to study the survival impact of FGS on resection margins in patients whom are simultaneously being studied for new chemotherapy regimens as this can confound results. Also, radio-, chemo-, and/or immunotherapy might affect the receptor overexpression [44] and vascularization of a tumor and therefore the binding capabilities of the imaging agent. The effect of neoadjuvant treatment on the expression of the biomarker of choice should be studied beforehand to avoid false-negative, or in some cases false-positive results.

In summary, challenges can be found in defining the incremental clinical value of using FGS and tumor-specific molecular imaging in particular. The gain of information provided by the spatial and biomolecular feedback of fluorescence guidance in the operating room will in some cases force clinicians to change treatment policy; the effect of which should be studied in order to establish new evidence-based decision models. New standards need to be set on trial design and reporting of results in order to successfully translate and implement FGS. Valuable examples can be found in the IDEAL framework describing the different stages of surgical innovations [45]. Integration of FGS with other non-invasive molecular imaging approaches [46] can empower precision cancer surgery, where accurate patient selection based on the tumor’s biology is the center of the personalized treatment. The field should continue its effort in studying new approaches to achieve the beforementioned. In many ways, collaboration between all the stakeholders in the process, from benchtop to bedside, seems to be the appropriate way to achieve this.

## Abbreviations

FDG-PET: Fluorodeoxyglucose positron emission tomography
CT: Computed tomography
FDA: Food and Drug Administration
FGS: Fluorescence-guided surgery
TS-FGS: Tumor-specific fluorescence-guided surgery
ICG: Indocyanine green
NIRF: Near-infrared fluorescence
EPR: Enhanced permeability and retention
CA19-9: Cancer antigen 19-9
CA125: Cancer antigen 125
PSA: Prostate-specific antigen
CEA: Carcinoembryonic antigen
CSE: Clinical significant event
SPECT: Single photon emission computed tomography
